# Human Infection with Eurasian Avian-like Influenza A(H1N1) Virus, China

**DOI:** 10.3201/eid1910.130420

**Published:** 2013-10

**Authors:** Da-Yan Wang, Shun-Xiang Qi, Xi-Yan Li, Jun-Feng Guo, Min-Ju Tan, Guang-Yue Han, Yan-Fang Liu, Yu Lan, Lei Yang, Wei-Juan Huang, Yan-Hui Cheng, Xiang Zhao, Tian Bai, Zhao Wang, He-Jiang Wei, Ning Xiao, Yue-Long Shu

**Affiliations:** National Insititute for Viral Disease Control and Prevention, China CDC. Beijing, People’s Republic of China (D.-Y. Wang, X.-Y. Li, J.-F. Guo, M.-J. Tan, Y. Lan, L. Yang, W.-J. Huang, Y.-H. Cheng, X. Zhao, T. Bai, Z. Wang, N. Xiao, H.-J. Wei, Y.-L. Shu);; Hebei Center for Disease Control and Prevention, Hebei Province, People’s Republic of China (S.-X. Qi, G.-Y. Han, Y.-F. Liu)

**Keywords:** influenza, surveillance, serology, avian-like influenza A(H1N1), viruses

**To the Editor:** We report a human infection with avian-like swine A(H1N1) influenza virus first identified through a surveillance system for influenza like illness (ILI) in mainland China. An influenza virus, isolated from a patient with ILI, was originally subtyped as influenza A(H1N1)pdm09 virus with a hemagglutination inhibition (HI) test, but it was identified as a Eurasian avian-like influenza A(H1N1) virus (EA-H1N1) by full genome sequencing on January 30, 2013. The virus was named A/Hebei-Yuhua/SWL1250/2012 (H1N1v) (HB/1250/12), according to the definition of the World Health Organization (*1*).

The case-patient was a 3-year-old boy who had symptoms of fever and sore throat; his highest body temperature was 38°C on December 9, 2012. He was brought for medical treatment to an influenza sentinel hospital in the city of Shijiazhuang in Hebei Province, China, on December 12. He recovered within a week without hospitalization and oseltamivir treatment. A throat swab specimen was collected and sent to the local Chinese Center for Disease Control and Prevention for virus isolation and characterization, according to the Guidelines of the Chinese National Influenza Surveillance Network. A retrospective investigation was conducted to identify the potential infection source and any other possible cases. The case-patient was previously healthy and had no history of close contact with animals (live or dead wild birds, poultry, and swine) within 2 weeks before the onset of symptoms, nor a history of travel. He lived with his sister and parents; all other family members did not develop influenza-like symptoms during the period of the investigation.

Sporadic human infections with swine influenza virus had been reported previously (*2*,*3*). Another case-patient, infected by EA-H1N1 influenza virus A/Jiangsu/1/2011(JS11) in early 2011, was reported (*4*,*5*). The genome sequences of the viruses isolated from the 2 case-patients showed high homology; the similarity of the polymerase basic protein 2 was 99.1%; of polymerase basic protein 1, 99.3%; of polymerase acidic protein, 98.9%; of hemagglutinin (HA), 99.1%; nucleocapsid protein, 99.1%; neuraminidase protein, 99.2%; matrix protein, 99.6% and of nonstructural protein, 99.2% (Global Initiative on Sharing Avian Influenza Data, GISAID, accession no.EPI301156–63 for JS11 and EPI438417–25 for HB/1250/12). The HB/1250/12 virus has the amino acids D (at site 190) and E (at site 225) within the HA protein, which are reported to be critical for enhancement of the HA affinity in binding to α-2,6–linked sialosides (*6*). The virus was resistant to amantadine and rimantadine with S31N (Ser→Asn) mutation in M2 gene, but was predicted to be susceptible to the neuraminidase inhibitor drugs oseltamivir and zanamivir on the basis of the neuraminidase gene.

HI test with ferret anti-serum against A(H1N1)pdm09 (CA09), seasonal H3N2(Vic11,BR10/07 and Perth09), classical swine subtype H1N1(NJ76), and the seasonal influenza subtype H1N1 viruses (BR59/07, SI06) showed that the HB/1250/12 virus is antigenically indistinguishable from NJ76 and CA09, but different from subtype H3N2 viruses (Vic11, BR10/07, and Perth09) and seasonal subtype H1N1 viruses(BR59/07, SI06) (Technical Appendix Table). These findings were consistent with results reported previously (*7*–*9*).

To estimate the susceptibility of human population to this virus, and to investigate whether seasonal trivalent inactivated influenza vaccine (TIV) could provide cross-protection, we collected serum samples from children, adults, and elderly adults, before and after 2012–2013 TIV vaccination, and the antibody against HB/1250/12 virus was tested by HI assay. The seroprotection antibody was defined as HI titers >40. Before vaccination, 28% of children (3–5 years) and 6.7% of adults (18–59 years) had HI titers >40, but elderly adults (>60 years) did not. Samples from 56% of children, 56.7% of adults, and 26.7% of elderly adults had HI titers >40 after TIV vaccination; however, a 4-fold antibody rise developed in <30% in any age group (Table). These results indicated that a proportion of cross-protective antibody against EA-H1N1 exists in children and adults, whereas elderly adults are the most susceptible to EA-H1N1 infection with no cross-protective antibody, the vaccination with TIV could not substantially improve the level of cross-reactive EA-H1N1 antibodies.

**Table:** Cross-reactive antibody response against avian-like influenza A(H1N1) virus in pediatric and adult recipients of seasonal trivalent inactivated influenza vaccines, China, 2013*†

Age group, y	Antigen	Increase ≥4, %‡		Geometric mean titer			%Titer ≥40			%Titer ≥160	
				Before vac.	After vac.		Before vac.	After vac.		Before vac.	After vac.
Children, n = 25	A/California/7/2009	60.0		21.1	121.3		44.0	84.0		16.0	60.0
3–5	HB/1250/12	24.0		12.8	25.7		28.0	56.0		0	24.0
Adults, n = 30	A/California/7/2009	70.0		14.8	156.3		26.7	76.7		0	60.0
18–59	HB/1250/12	6.7		8.1	31.7		6.7	56.7		0	23.3
Elderly adults, n = 30	A/California/7/2009	46.7		10.5	52.8		10.0	53.3		0	30.0
>60	HB/1250/12	26.7		5.7	11.5		0	26.7		0	6.7

*Vac., vaccination.  †All children received 2 doses of vaccine with an interval of 1 month. The composition of the trivalent vaccine were A/Christchurch/16/2010(NIB-74xp) (A/California/7/2009-like), A/Victoria/361/2011 (H3N2)IVR-165, and B/Hubei-Wujiagang/158/2009. Serum samples were obtained from vaccine recipients living in northern (children) and southern (adults and elderly adults) China. ‡Increase in antibody titer.

Antisera from hyperimmune sheep are usually used for influenza virus typing and subtyping, the CA09 sheep antisera reacted well with the HB/1250/12 virus (Technical Appendix Table). This is the reason why the local Chinese Center for Disease Control and Prevention originally subtyped HB/1250/12 as A(H1N1)pdm09 virus. Such avian-like H1N1 virus could be missed with regular HI test. In addition, a large proportion of swine influenza infection cases are mild and even asymptomatic (*2*); thus, the human infections with swine influenza virus may have been underestimated in China.

This is the first human case of EA-H1N1 infection identified through the national ILI surveillance network in China, indicating that the influenza surveillance network not only plays a critical role in monitoring the seasonal influenza circulation and the vaccine virus selection, but also is useful for early detection of novel influenza viruses with pandemic potential. This study also highlighted the value of, and urgent demand for, a cost-effective sequencing platform on routine influenza surveillance for pandemic preparedness.

Technical Appendix Table showing cross-reactive antibody response against avian-like influenza A(H1N1) virus in pediatric and adult recipients of seasonal trivalent inactivated influenza vaccines, China, 2013
