# Supplementary material for: Human Infection with Eurasian Avian-like Influenza A(H1N1) Virus, China
Source: Emerg Infect Dis. 2013 Oct;19(10):1709–11. doi: 10.3201/eid1910.130420 (PMC3810748; doi:10.3201/eid1910.130420)
Supplement: Technical Appendix — Table showing cross-reactive antibody response against avian-like influenza A(H1N1) virus in pediatric and adult recipients of seasonal trivalent inactivated influenza vaccines, China, 2013 [file 13-0420-Techapp-s1.pdf]

Article DOI: http://dx.doi.org/10.3201/eid1910.13420

Human Infection with Eurasian Avian-like Influenza A(H1N1) Virus, China

Technical Appendix

Technical Appendix Table. Cross-reactive antibody response against avian-like influenza A(H1N1) virus in pediatric and adult recipients of seasonal trivalent inactivated influenza vaccines, China, 2013*†

Age group, y	Antigen	Increase ≥4, %‡	Geometric mean titer		Titer ≥40, %		Titer ≥160, %	
			Before vac	After vac	Before vac	After vac	Before vac	After vac
Children, n = 25	A/California/7/2009	60.0	21.1	121.3	44.0	84.0	16.0	60.0
3–5	HB/1250/12	24.0	12.8	25.7	28.0	56.0	0	24.0
Adults, n = 30	A/California/7/2009	70.0	14.8	156.3	26.7	76.7	0	60.0
18–59	HB/1250/12	6.7	8.1	31.7	6.7	56.7	0	23.3
Elderly adults, n = 30	A/California/7/2009	46.7	10.5	52.8	10.0	53.3	0	30.0
≥60	HB/1250/12	26.7	5.7	11.5	0	26.7	0	6.7

*Vac, vaccination.
†All children received 2 doses of vaccine with an interval of 1 month. The composition of the trivalent vaccine were A/Christchurch/16/2010(NIB-74xp) (A/California/7/2009-like), A/Victoria/361/2011 (H3N2)IVR-165, and B/Hubei-Wujiagang/158/2009. Serum samples were obtained from vaccine recipients living in northern (children) and southern (adults and elderly adults) China.
‡Increase in antibody titer.
